# Ninth International Medical Congress

**Published:** 1887-09

**Authors:** 


### NINTH INTERNATIONAL MEDICAL CONGRESS.

HELD IN WASHINGTON, D. C., SEPT. 5, 6, 7, 8, 9 AND 10, 1887.

The Congress assembled in Albaugh's Opera House and was formally opened at 11 a. m., Sept. 5, by his Excellency Grover Cleveland, President of the United States. Dr. Henry H. Smith, of Philadelphia, chairman of the executive committee next announced the officers of the Congress, all of whom were formally elected. The Secretary-General, Dr. J. B. Hamilton made his report, reviewing the work of preparation, and Dr. A. P. Y. Garnett, of Washington, chairman of the committee of arrangements announced the arrangements for the social entertainment of the members of the Congress and their families. Hon. Thos. F. Bayard, Secretary of State, delivered the address of welcome, which was responded to by representatives of each country, as follows: Dr. Wm. H. Lloyd, of the Royal Navy, for England, Dr. Leon Le Fort, of France, Prof. Unna for Germany, Prof. Semmola for Italy, Dr. Charles Reyher for Russia. Dr. L. A. Sayre, of New York, occupied the chair, whileDr. Davis, the President, delivered his address. Dr. Davis' address is said to have been a very brilliant effort. It is a matter of regret that we cannot produce it entire,—but the fact is, the International Congress is too big a thing for any Journal to report in full, and it is difficult to select any part for presentation, when all is so good.

We cannot refrain from reproducing the following extract, however,—if for nothing else, to get in his excellent hit at his old enemy *homœopathy*, but mainly to show how broad and comprehensive is his view of the field of medicine:

"The living human body, the chief object of your solicitude, not only combines in itself the greatest number of elementary substances and the most numerous organs and varied functions, so attuned to harmonious action as to illustrate the operation of every law of physics, every known force in nature, and every step in the development of living matter, from the simple aggregation of protoplasm constituting the germinal cell to the full-grown man, but it is placed in appreciable and important relations with the material objects and immaterial forces existing in the world in which he lives.

Hence a complete study of the living man, in health and disease involves a thorough study, not only of his structure and functions but more or less of every element and force entering into the earth, the air, and the water with which he stands in constant relation.

The medical science of to-day, therefore, embraces not only a knowledge of the living man but also of such facts, principles, and materials gathered from every other department of human knowledge as may increase your resources for preventing or alleviating his suffering and of prolonging his life.

The time has been when medical studies embraced little else than the fanciful theories and arbitrary dogmas of a few leading minds, each of which became for the time the founder of a sect or so-called school of medicine, with his deciples more or less numerous. But with the development of general and analytical chemistry, of the several departments of natural science, of a more practical knowledge of physics, and the adoption of inductive processes of reasoning, the age of theoretical dogmas and of medical sects blindly following some more plausible leader passed away, leaving but an *infinitesimal* shadow yet visible on the medical horizon.

That the Congress was a big success, there can be no doubt. The *Register* says there were more members registered and in attendance the first day than at Copenhagen (last Congress) the entire week, and that was considered a good one. Two hundred and fifty sat at one time in the section of Gynecology.

It was our intention to report part of the proceedings, but as there is such an immense amount of it, and as all the Weekly journals will be out with it before we can publish it, we have not even attempted a synopsis. It is thought the section work, alone, will fill several large volumes. We will, however, from time to time, publish abstracts of the best papers read.

